# 四氧化三铁/金属有机框架复合材料的制备策略及其在样品前处理中的应用进展

**DOI:** 10.3724/SP.J.1123.2026.04033

**Published:** 2026-11-08

**Authors:** Yaxin LI, Minghua LU

**Affiliations:** 河南大学化学与分子科学学院，河南 开封 475004; College of Chemistry and Molecular Sciences，Henan University，Kaifeng 475004，China

**Keywords:** 四氧化三铁, 金属有机框架, 磁性纳米复合材料, 样品前处理, 磁性固相萃取, magnetite （Fe_3_O_4_）, metal-organic frameworks （MOF）, magnetic nanocomposites, sample pretreatment, magnetic solid phase extraction

## Abstract

四氧化三铁/金属有机框架复合材料结合了四氧化三铁纳米粒子的超顺磁性和金属有机框架的高比表面积、可调孔道，成为样品前处理领域的新型材料。本文系统总结了一锅法、原位生长法、后合成修饰法及逐层自组装法4种制备策略，分析了各方法的界面结合机制、结构可控性及实际应用性能。在此基础上，详细评述了该材料在食品、生物样品、环境水体、化妆品及法医毒理学等领域的前处理应用进展。针对当前研究中存在的金属有机框架负载不均匀、循环使用次数有限、复杂基质中长期性能验证不充分等问题，提出了未来应着力发展合成简便、结构可控、可重复使用的高选择性吸附剂，并推动前处理与检测仪器的在线联用，为相关研究提供参考。

## Abstract (en)

Sample pretreatment is a critical step in analytical chemistry，directly affecting the accuracy and reliability of analytical results. Traditional methods such as liquid-liquid extraction and solid phase extraction suffer from high organic solvent consumption，complicated procedures，and limited selectivity. Magnetic solid phase extraction （MSPE），as a novel pretreatment technology，has attracted extensive attention due to its simple operation，rapid separation，and minimal solvent consumption. MSPE involves adsorption，magnetic separation，washing，and elution steps，where magnetic adsorbents are quickly separated from sample matrices under an external magnetic field，eliminating centrifugation or filtration steps. Among various magnetic materials，Fe_3_O_4_ nanoparticles are the most commonly used magnetic carriers due to their low cost，high magnetic responsiveness，and good biocompatibility. However，Fe_3_O_4_ nanoparticles have inherent drawbacks such as small specific surface area，limited active sites，and poor adsorption selectivity. Metal-organic frameworks （MOFs），formed by self-assembly of metal ions with organic ligands，possess high specific surface area，tunable pore structure，rich surface chemistry，and excellent stability. MOFs have shown great potential in gas adsorption，catalysis，sensing，and sample pretreatment as excellent adsorbents. However，MOF materials face challenges such as difficult separation and poor reusability. The combination of Fe_3_O_4_ and MOFs leverages their synergistic advantages. MOFs provide abundant active sites for adsorption，while Fe_3_O_4_ endows the composite with rapid magnetic separation capability. This composite solves the recovery difficulty of MOFs and overcomes the limitations of Fe_3_O_4_ in adsorption capacity and selectivity. Magnetite/metal-organic framework （Fe_3_O_4_/MOF） composites combine the superparamagnetism of Fe_3_O_4_ with the high specific surface area and tunable pore structure of MOFs，emerging as promising materials for sample pretreatment. This review systematically summarizes four main preparation strategies： one-pot method，in-situ growth method，post-synthetic modification method，and layer-by-layer assembly method. The interfacial binding mechanisms，structural controllability，and practical application performance of each method are analyzed in detail. On this basis，the application progress of Fe_3_O_4_/MOF composites in sample pretreatment is comprehensively reviewed，covering food analysis，biological samples，environmental water monitoring，cosmetics testing，and forensic toxicology. Despite their promising prospects，different preparation methods yield materials with varying MOF loading uniformity and cyclic stability. Long-term performance validation in complex matrices remains insufficient. Current research issues include uneven MOF loading，harsh preparation conditions，limited reusability cycles，and inadequate performance verification. Future research should focus on developing adsorbents with simple synthesis，controllable structure，high selectivity，and good reusability. Promoting online coupling of sample pretreatment with detection instruments will also be a key step toward practical application. This review provides a reference for related fields by systematically summarizing the preparation strategies and application progress of Fe_3_O_4_/MOF composites.

样品前处理是分析化学中的关键步骤，直接关系到分析结果的准确性、精密度和可靠性^［1，2］^。在环境监测、食品安全检验、生物医药分析等领域，待测样品往往基质复杂，共存干扰物质众多，目标分析物浓度通常处于痕量或超痕量水平，这对样品前处理技术提出了极高的要求^［3，4］^。传统的样品前处理方法，如液液萃取、固相萃取等，虽然已在实际分析中得到了广泛应用，但仍存在有机溶剂消耗大、步骤烦琐、耗时长、选择性有限等局限性^［5-7］^。因此，发展高效、快速、环境友好的新型样品前处理技术成为分析化学领域的研究热点。

磁性固相萃取（magnetic solid phase extraction，MSPE）作为一种新型样品前处理技术，凭借操作简便、分离快速、溶剂用量少等优势受到广泛关注^［8］^，该技术通过吸附、磁分离、洗涤和洗脱等步骤完成样品前处理。首先将磁性吸附剂分散于样品溶液中，通过吸附作用富集目标分析物。然后施加磁场使吸附剂与样品基质快速分离，省去离心或过滤步骤。分离后的吸附剂用少量溶剂去除杂质，再用适当洗脱溶剂将目标物回收，最后进行仪器分析。与容易堵塞的传统固相萃取柱相比，MSPE操作更便捷、处理时间更短，尤其适合复杂基质样品的分析^［9，10］^。

磁性纳米材料具有超顺磁性，是实现MSPE技术的关键载体^［11］^。在外加磁场的作用下，磁性材料能够与样品溶液快速分离，省去了离心、过滤等一系列烦琐的操作，简化了前处理的过程^［12］^。各类磁性材料当中，四氧化三铁（Fe_3_O_4_）纳米粒子因为制备工艺简单、成本低、磁响应性高、生物相容性好，被用作磁分离技术中最常用的主要载体^［13，14］^。但是该材料的比表面积较小、表面活性位点少、吸附选择性差等缺点限制了它在复杂样品前处理中的使用^［15-17］^。金属有机框架（metal-organic frameworks，MOFs）是近年来发展起来的一种新型多孔晶体材料，由金属离子或者簇和有机配体通过配位键自组装形成，具有高比表面积、可调孔道结构、表面化学性质丰富、稳定性好等特点^［18-21］^。自20世纪90年代开始，MOFs在气体吸附和分离、催化、传感、药物递送等各个领域都具有很大的应用前景^［22-25］^。在样品前处理方面，MOFs可以作为很好的吸附剂来富集各种目标物^［26-29］^。但是MOF纳米材料在实际应用中存在分离回收难、重复使用性差等问题^［30-32］^。将Fe_3_O_4_和MOFs复合，可以发挥二者的协同作用。MOFs有高比表面积和规则孔道结构，可给吸附提供丰富的活性位点^［33，34］^，Fe_3_O_4_超顺磁性让材料具有快速磁分离的能力^［35，36］^。这种复合材料既解决了MOF回收难的问题，又克服了Fe_3_O_4_吸附容量低、选择性不足的缺陷，将“分离”与“富集”功能融为一体^［37］^。

虽然Fe_3_O_4_/MOF复合材料在样品前处理方面有很好的发展前景，但是不同的制备方法得到的材料在MOF负载均匀性、结构可控性、循环稳定性等方面存在差别，在复杂的基质中长期性能验证也不够充分^［38-41］^。因此，对Fe_3_O_4_/MOF复合材料的制备方法进行系统总结，分析各种制备方法的特点及应用范围，对开发高效、绿色的新型前处理吸附材料具有参考价值。本文将阐述一锅法、原位生长法、后合成修饰法和逐层自组装法4种主要制备策略，并评述该材料在食品、生物医药、环境、化妆品及法医毒理学等领域的前处理应用进展。

## 1 Fe_3_O_4_/MOF复合材料的制备方法

Fe_3_O_4_/MOF复合材料的制备方法很多，不同的方法在界面结合机制、结构可控性、功能可调性和实际应用性能等方面各有特点。根据构筑路径的不同可以分为一锅法、原位生长法、后合成修饰法和逐层自组装法4种。一锅法操作简单、周期短，适合于快速制备；原位生长法可以形成紧密结合的核壳结构，壳层厚度可控制；后合成修饰法在保持MOF骨架完整性的基础上加入特定的功能基团，可提高选择性识别能力；逐层自组装法则通过逐层交替沉积实现厚度和组成的精确调控。合理选择制备方法，是实现Fe_3_O_4_/MOF复合材料高效样品前处理应用的关键。

### 1.1 一锅法

一锅法是制备Fe_3_O_4_/MOF复合材料的一种简便高效的合成方法。该方法是将预合成的Fe_3_O_4_和金属盐、有机配体、溶剂直接混合，采用一步水热或者溶剂热反应，使MOF在Fe_3_O_4_表面或周围生长，形成Fe_3_O_4_/MOF复合材料。这种方法操作简便，合成过程中不用额外分离中间产物，重现性好且易于放大。通过改变反应条件来控制MOF的负载量、晶体尺寸以及磁响应性，适合废水处理、催化降解和固相萃取等对吸附容量要求较高的场合^［41，42］^。如图1所示，Wang等^［43］^采用一锅法，将Fe_3_O_4_纳米粒子、ZrCl_4_和2-硝基对苯二甲酸同时分散于*N*，*N*-二甲基甲酰胺中，120 ℃反应24 h，一步制备出Fe_3_O_4_@UiO-66-NO_2_纳米复合材料。MOF直接包覆在Fe_3_O_4_表面上，不需要中间的分离步骤，操作简单高效。所得材料比表面积310 m^2^/g，饱和磁化强度14.99 emu/g。与纯UiO-66-NO_2_相比，该复合材料可以在6 min内完全吸附5种磷酸糖代谢物，并且可以进行磁分离来实现快速富集。该材料可以用来检测血清中的磷酸糖，检出限低至0.001 ng/mL。类似地，Chong等^［44］^将Fe_3_O_4_纳米粒子直接加入到铜基金属有机框架（Cu-MOF）前驱体溶液中，在60 ℃下反应4 h，得到Cu-MOF@Fe_3_O_4_磁性复合材料，光催化去除ReO_4_⁻的效率由原来的85%提高到了97%，可以用于处理含有铼、锝的放射性废水。为了缩短合成时间并提高比表面积，引入了超声辅助一锅法。Abdtawfeeq等^［45］^将Fe_3_O_4_、MoCl₅和吡啶-2，6-二甲酸在乙酸/水混合溶剂中80 ℃搅拌15 min，再用370 W超声反应20 min，制得比表面积1 500 m^2^/g的Fe_3_O_4_/Mo-MOF。超声辅助可以大大缩短合成时间，且获得的材料形貌均匀。该材料作为非均相催化剂，在40 ℃水/乙醇混合溶剂中催化醛、丙二腈和巴比妥酸衍生物的3组分反应，合成新型吡喃并［2，3-d］嘧啶衍生物，产率超过93%，通过磁铁快速分离回收，重复使用6次，活性没有明显下降。

**图1:**
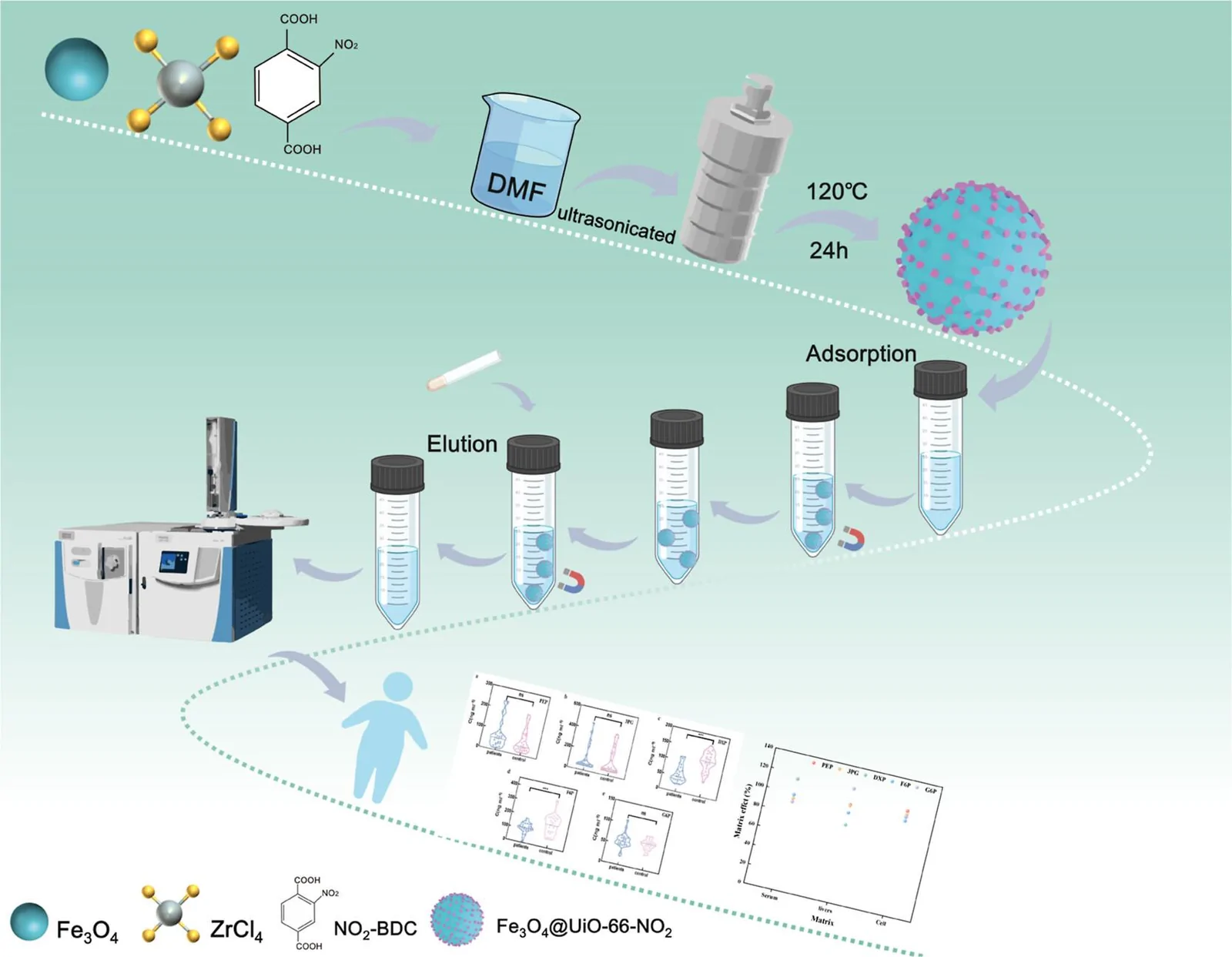
一锅法制备Fe_3_O_4_@UiO-66-NO_2_及其用于血清中磷酸糖代谢物的磁性固相萃取^［43］^ DMF： *N*，*N*-dimethylformamide； NO_2_-BDC： 2-nitroterephthalic acid.

### 1.2 原位生长法

原位生长法是制备核壳结构Fe_3_O_4_/MOF复合材料的常用策略之一，也是目前应用较为广泛的方法。该方法以Fe_3_O_4_纳米粒子为载体，经表面羧基、氨基、二氧化硅或聚合物层修饰，增强与MOF前驱体的界面亲和力。在水热或溶剂热条件下，MOF晶体直接在磁核表面成核生长，得到结构稳定的核壳型复合材料^［46］^。该法制备的MOF壳层与磁核结合紧密，结构完整，可以通过调节反应时间、温度、前驱体浓度精确控制壳层厚度、晶体形貌及孔道特征^［47］^。与一锅法随机混合不同的是，原位生长法依靠磁核表面官能团来引导MOF优先成核，形成化学键合的完整核壳结构，界面结合牢固，稳定性好，更适用于多次循环使用。最基本的方法是依靠磁核表面官能团促进MOF沉积，如图2所示，为提高合成效率，Xiang等^［48］^采用微波辅助原位生长，使羧基化Fe_3_O_4_与*γ*-环糊精、氢氧化钾（KOH）快速反应，形成环糊精基金属有机框架（cyclodextrin-based metal-organic frameworks，CD-MOFs）壳层，300 ℃热解后得到空心磁性微立方体，对Cd（Ⅱ）、Pb（Ⅱ）、Cu（Ⅱ）的吸附容量分别为129.9、151.1、107.0 mg/g。Feng等^［49］^以聚多巴胺（polydopamine，PD）层为锚定位点，通过由5，10，15，20-四（4-羧基苯基）卟啉（5，10，15，20-tetrakis（4-carboxyphenyl）porphyrin，TCPP）和1，4-苯二硼酸（1，4-phenylenebisboronic acid，BA）组成的双配体，诱导硼酸修饰的卟啉基Zr-MOF在Fe_3_O_4_表面原位生长，制得Fe_3_O_4_@PD@BA-Zr-MOF。该材料具有硼酸亲和、金属氧化物亲和与亲水作用，对顺式二醇核苷有较好的选择性富集效果，对胞苷、尿苷、鸟苷和腺苷的检出限为0.002~0.005 μg/mL，重复使用5次后萃取效率基本不变。在此基础上，Li等^［50］^又对双配体策略进行了扩展，在羧基化的Fe_3_O_4_表面分别吸附Zr^4+^、Eu³^+^，再加入含有氨基和硼酸基的双配体，原位生长得到核壳结构的Fe_3_O_4_-Zr/Eu MOF。该材料中Zr^4+^催化棉酚（Gsp）形成硼酸酯和席夫碱，引发Eu³^+^荧光猝灭，实现灵敏检测，检出限为0.39 nmol/L，同时Fe_3_O_4_核赋予快速磁分离能力，对棉酚吸附量达157.21 mg/g。

**图2:**
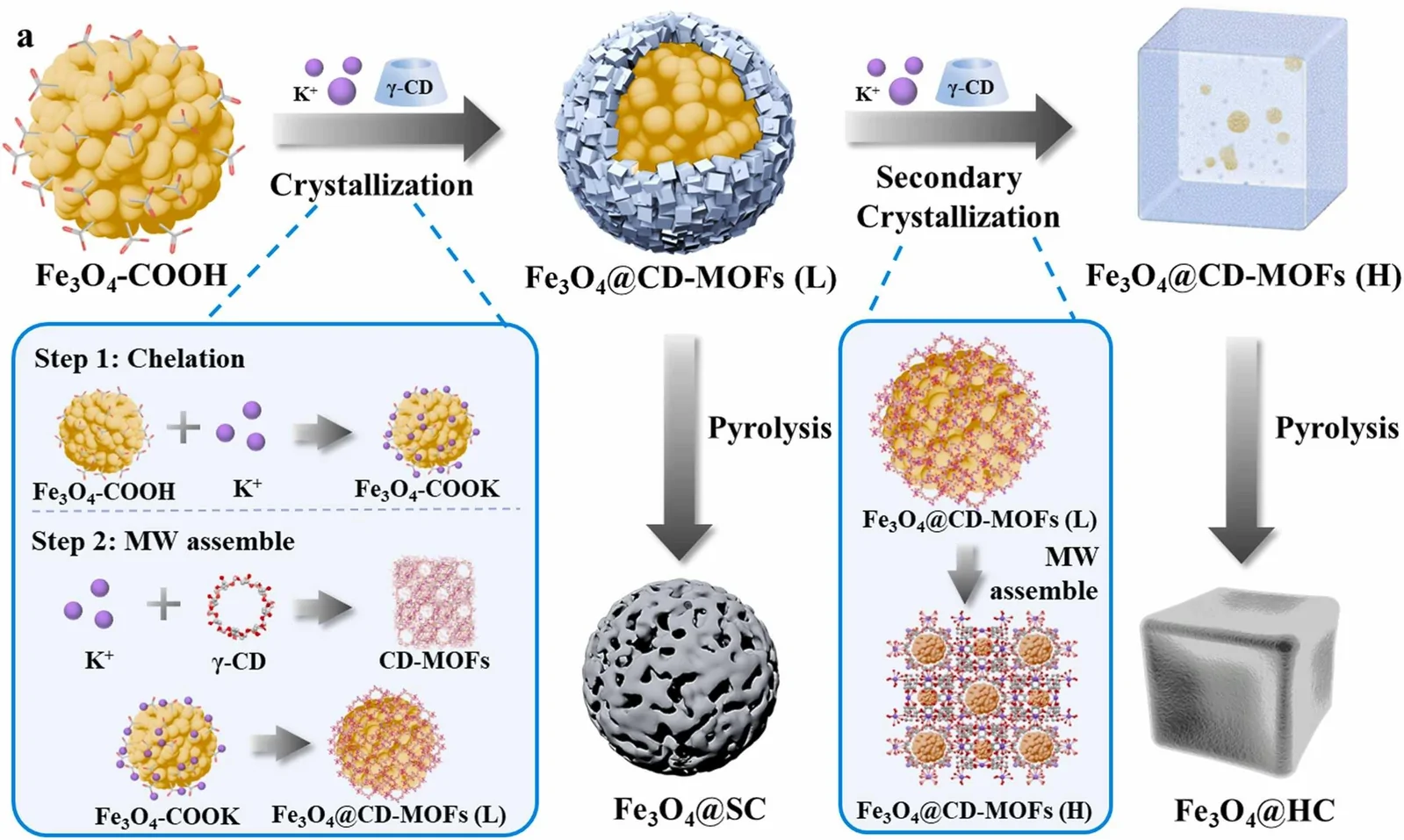
微波辅助原位生长法制备Fe_3_O_4_@CD-MOFs及其热解产物的示意图^［48］^ CD： cyclodextrin； MW： microwave； SC： spherical carbon； HC： hollow carbon microcube； L： low loading； H： high loading.

### 1.3 后合成修饰法

后合成修饰法是在Fe_3_O_4_/MOF复合材料表面引入功能基团，提高材料的选择性识别和特异性吸附能力。制备好的基础复合材料中，MOF骨架上未被配位的金属位点或者有机配体上的氨基、羧基、羟基等可反应基团，可以和硫醇、胺类、聚合物或者生物活性分子发生共价反应、配位作用或者氢键相互作用，从而达到表面或者孔道定向修饰的目的^［51］^。这种修饰方式在保持MOF原始孔结构和晶体骨架完整性的基础上引入更多的功能位点，提高对目标分析物的亲和力和选择性，可以针对目标物官能团、极性或者尺寸进行设计，适合复杂样品中痕量组分的定向富集^［52，53］^。根据修饰目的，后合成修饰法可分为靶向特定官能团修饰和生物分子修饰两种。如图3所示，Li等^［54］^先合成Fe_3_O_4_@Hf/Ti-MOF-NH_2_，利用壳层氨基与*O*-甲基异脲在pH 11、60 ℃条件下反应24 h，通过亲核取代转化为胍基，制得Fe_3_O_4_@Hf/Ti-MOF-Gua。该材料利用双金属对磷酸根有亲和力、胍基有亲水性的特点，使得磷酸肽的富集灵敏度达到20 fmol，在复杂体系中的回收率为85.4%。Yazdiamiri等^［55］^先将UiO-66-NH_2_用戊二醛和腺苷功能化，再与Fe_3_O_4_在*N*，*N*-二甲基甲酰胺中室温搅拌24 h复合，得到的材料对亚甲蓝的吸附容量为62.5 mg/g，可以催化合成四氢苯并吡喃衍生物（产率大于94%），具有废水净化和有机催化的功能。除了氨基、胍基等功能化修饰外，利用MOF壳层中的羟基进行磷酸化后合成修饰也是提高吸附性能的有效方法。Zhu等^［56］^先构建了多层核壳结构Fe_3_O_4_@MnO_2_@UiO-66-（OH）_2_（FMU），其中UiO-66骨架上的自由羟基作为反应位点。将FMU和植酸在常温下搅拌24 h，通过酯化反应将磷酸基团共价接枝到MOF壳层上，得到FMU-P。该材料对U（Ⅵ）的吸附容量高达697.78 mg/g（pH 6），远超未修饰的FMU（367.16 mg/g）。磷酸基团（P=O和P-O）与铀酰离子的内层表面络合和静电吸引是吸附增强的主要原因。该Fe_3_O_4_@MnO_2_核给材料带来优异的磁分离性能，经过5次循环之后吸附效率仍然大于90%，对含铀废水的处理具有潜力。

**图3:**
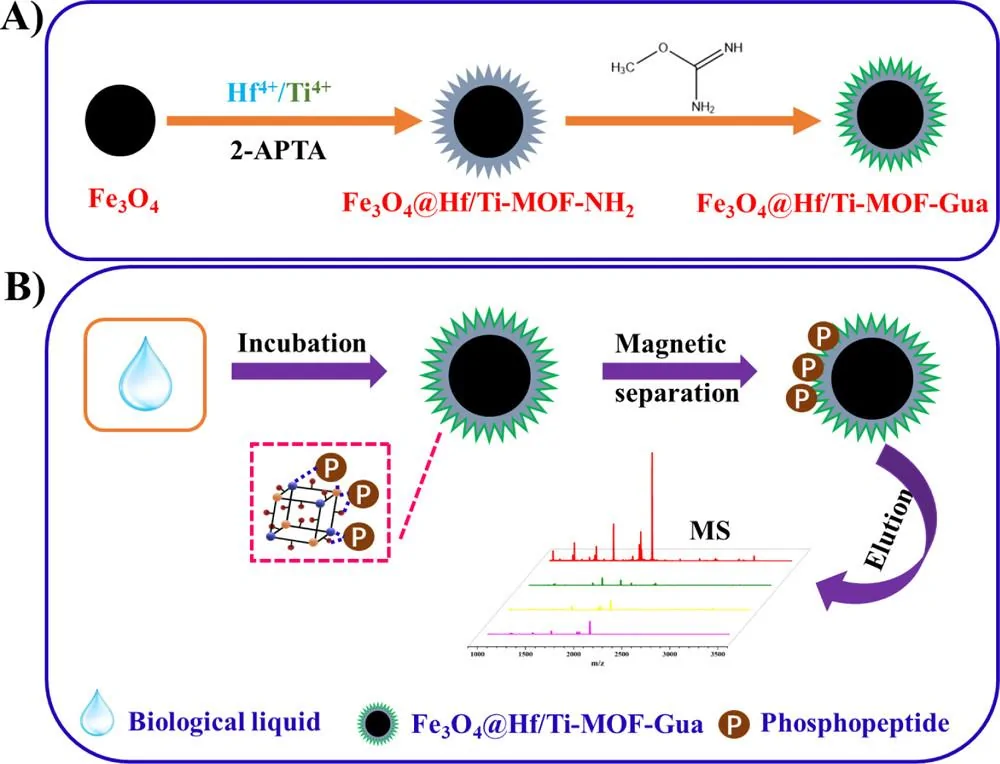
后合成修饰法制备Fe_3_O_4_@Hf/Ti-MOF-Gua用于磷酸肽富集^［54］^ 2-APTA： 2-aminoterephthalic acid； Gua： guanidino group； MS： mass spectrometry.

### 1.4 逐层自组装法

逐层自组装法可以精确地控制MOF壳层的结构和组成。该方法利用静电吸引、氢键或者配位等相互作用，在磁核表面交替沉积MOF壳层。与热力学自发驱动的自组装过程不同的是，逐层自组装一般要依靠超声、离心、pH调节等外力来驱动沉积过程，可以实现多层或多组分结构的精确控制^［57，58］^。该种方法可以对材料表面性质进行精细调节，但是操作过程比较烦琐，制备时间较长^［59］^。如图4所示，我们课题组^［60］^用带正电的Fe_3_O_4_和带负电的多元MOF（95.7% BTB/MOF-525，其中BTB为1，3，5-三（4-羧基苯基）苯（1，3，5-tris（4-carboxyphenyl）benzene））超声混合，利用正负电荷吸引快速成壳，在30 s内完成自组装。该材料对双酚F、A、B、AF的最大吸附容量分别为275.1、233.6、302.1和277.2 mg/g，富集因子为242~295，检出限低至0.067~0.167 ng/mL，21次循环后回收率仍超过80%，为环境水样中痕量双酚类污染物的高效吸附和快速磁分离提供了可行策略。Karaket等^［61］^用聚苯胺（PANI）-聚苯乙烯磺酸（PSS）、聚酰胺-胺树枝状聚合物（PAMAM）和氧化石墨烯量子点（QGO）进行静电作用逐层自组装，最后加入Fe_3_O_4_超声复合得到Fe_3_O_4_@PANI-PSS/PAMAM-QGO。树枝状聚合物、量子点的加入使得*π-π*堆积和疏水作用增强，使材料对多环芳烃的富集因子达到185，可以重复使用10次以上，适用于环境水样中痕量多环芳烃的检测。Huang等^［62］^利用层层沉积构建多组分壳层，先将Fe_3_O_4_纳米颗粒和Zn（NO_3_）_2_、2‑甲基咪唑混合，超声作用下形成ZIF‑8内层。随后向反应体系中加入Co（NO_3_）_2_，利用相同配体继续配位生长ZIF‑67外层，最终得到Fe_3_O_4_@ZIF‑8@ZIF‑67。相比于单层Fe_3_O_4_@ZIF-8，这种双MOF壳层的协同作用大大提高了吸附性能，对刚果红的去除率由原来的73.3%提高到99.4%，对四环素的吸附容量也达到了356.1 mg/g。

**图4:**
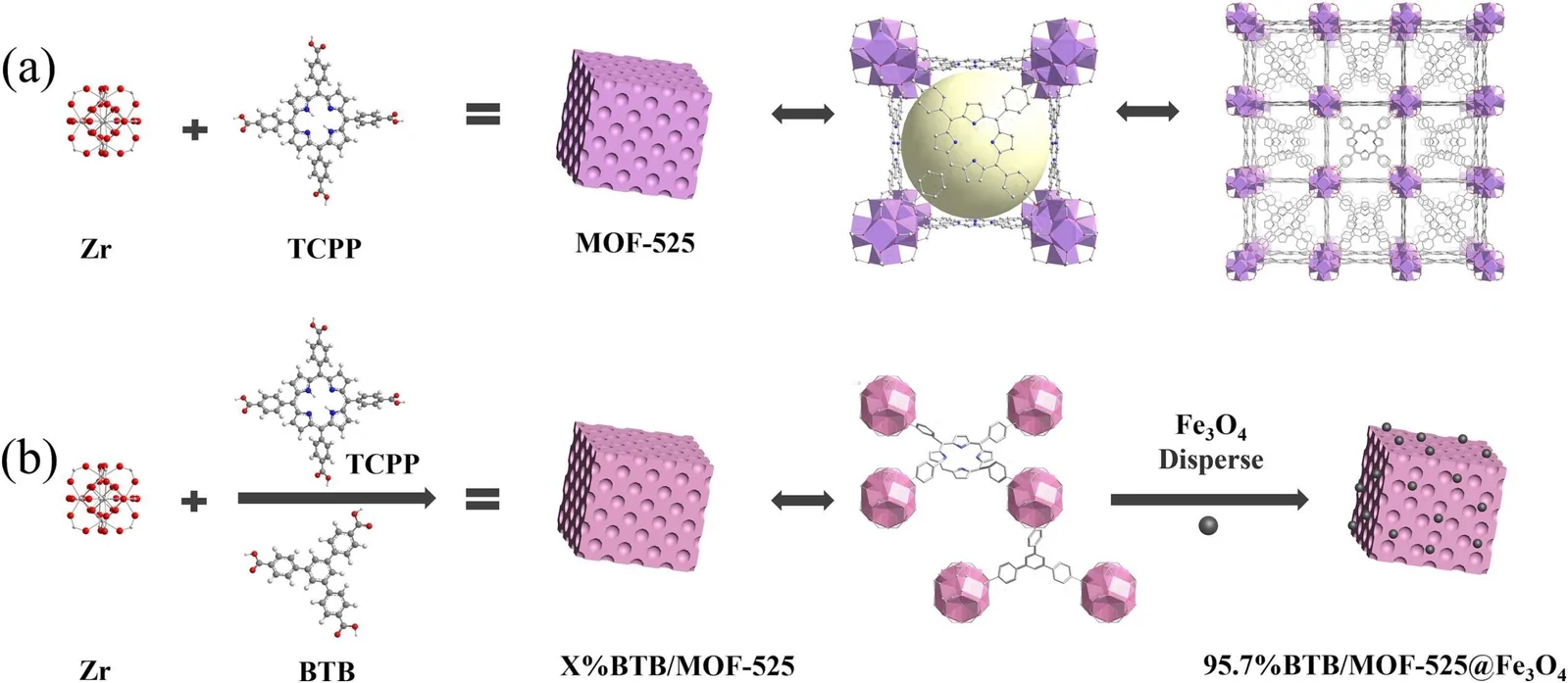
静电自组装法制备Fe_3_O_4_/多元MOF复合材料用于双酚类污染物的快速磁分离检测^［60］^ TCPP： 5，10，15，20-tetrakis（4-carboxyphenyl）porphyrin； BTB： 1，3，5-tris（4-carboxyphenyl）benzene.

## 2 Fe_3_O_4_/MOF复合材料在样品前处理中的应用

相比于传统吸附剂，Fe_3_O_4_/MOF复合材料能够在复杂基质中有效富集目标分析物，并可通过磁分离快速回收，从而显著缩短样品前处理时间、减少有机溶剂消耗。目前，Fe_3_O_4_/MOF复合材料已广泛应用于多个领域：在食品安全方面，用于抗生素、双酚类物质、农药等有害残留物的富集检测；在生物医药领域，实现了对血浆、尿液等生物样品中药物分子及低丰度内源性生物标志物的富集与高灵敏检测；在环境分析中，用于水体中重金属离子的去除与定量检测。此外，该材料在化妆品安全及法医毒理学领域，也已用于相关复杂基质的前处理与分析。

### 2.1 食品领域

食品样品基质复杂，痕量污染物浓度低，对前处理技术的富集效率和选择性要求高^［63-65］^。传统方法由于操作复杂、溶剂用量大、基质干扰严重等原因不能满足快速灵敏的检测要求^［66］^。Fe_3_O_4_/MOF复合材料具有高比表面积、可调孔道和快速磁分离能力，可以利用多种非共价作用来捕获目标物，在短时间内完成吸附剂与食品基质的分离^［67，68］^。目前该材料已经被用在各种食品样品中多种痕量污染物的富集和净化，提高了分析方法的灵敏度、回收率以及操作的便捷性^［69］^。如图5所示，Lv等^［70］^合成了UiO-66-NH_2_功能化的聚多巴胺（PDA）修饰磁性多壁碳纳米管（MWCNTs）复合材料（Fe_3_O_4_@MWCNTs@PDA@UiO-66-NH_2_），利用氨基功能化和碳纳米管的加入来提高吸附剂对目标物的萃取效率。采用MSPE模式富集牛奶和蜂蜜中的16种喹诺酮类药物，结合超高效液相色谱-串联质谱法（UPLC-MS/MS）检测，检出限为0.012~0.690 μg/kg。为了提高吸附剂的结构稳定性，Zhang等^［71］^将UiO-66碳化后和海藻酸钠（SA）、磁性颗粒复合，制备出具有多级孔结构的Fe_3_O_4_/UiO-66-C/SA磁性复合微球。该材料用于茶饮料和环境水中双酚类物质的MSPE富集和去除，结合高效液相色谱-荧光检测法（HPLC-FLD）检测，检出限低至0.022~0.054 ng/mL。多级孔结构设计加快了传质过程，也提高了材料的机械强度和重复使用性能。除了结构优化之外，萃取速度和操作简便性也是评价前处理方法实用性的指标。Jiang等^［72］^采用原位生长法制备了Fe_3_O_4_-BUT-18复合材料，用于食用油中黄曲霉毒素的快速检测。该材料比表面积为814.51 m^2^/g，在超声辅助下1 min内完成吸附，磁分离后用含0.1%甲酸的乙腈洗脱，结合液相色谱-串联质谱（LC-MS/MS）同时检测4种黄曲霉毒素（B_1_、B_2_、G_1_、G_2_），检出限为0.1~0.4 μg/kg，可重复使用3次。该方法在吸附容量、萃取效率、灵敏度和使用寿命等方面取得了较好的平衡。

**图5:**
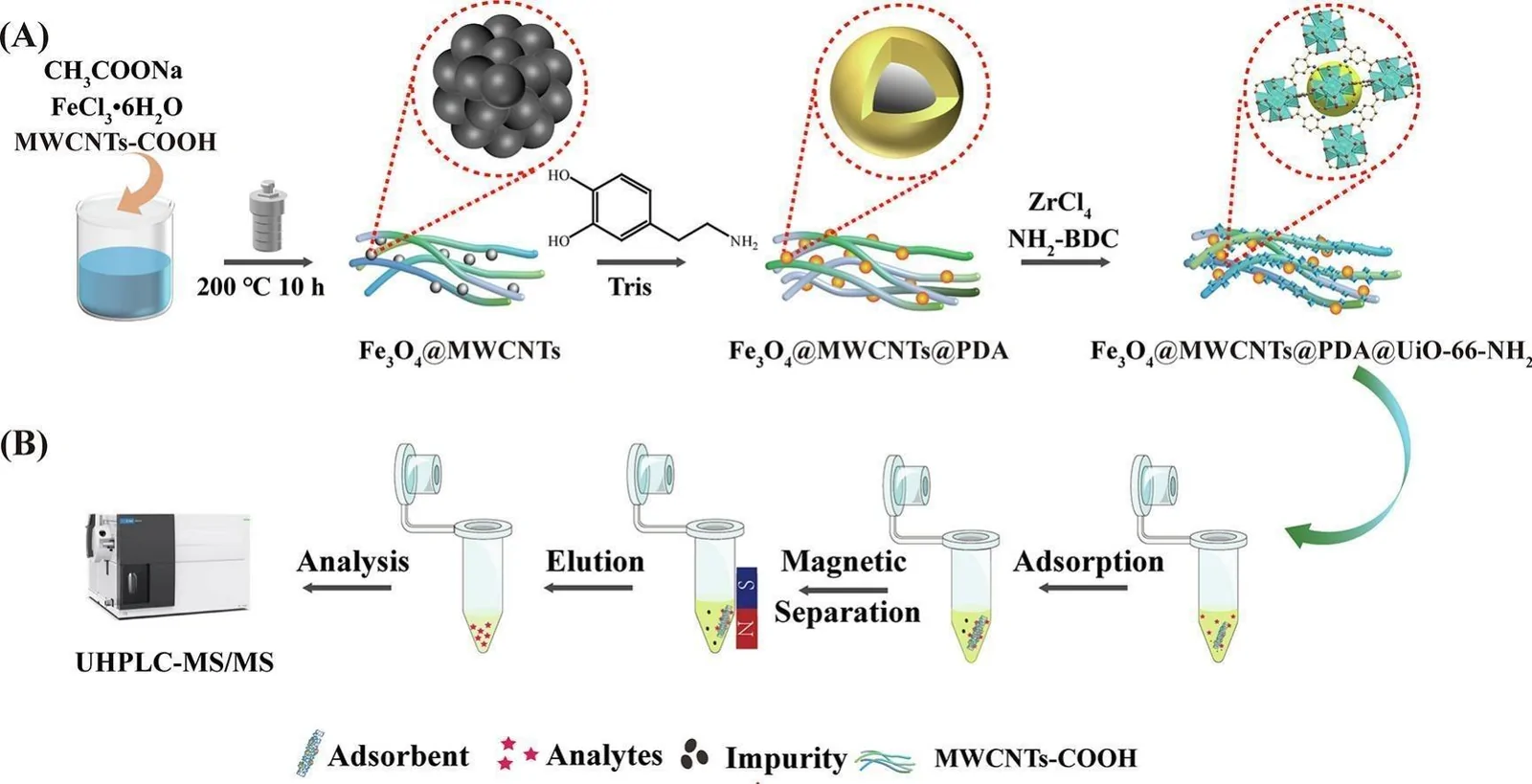
Fe_3_O_4_@MWCNTs@PDA@UiO-66-NH_2_磁性复合材料的制备及其用于牛奶和蜂蜜中16种喹诺酮类药物磁性固相萃取的示意图^［70］^ MWCNTs-COOH： carboxylated multi-walled carbon nanotubes； Tris： tris（hydroxymethyl）aminomethane； NH_2_-BDC： 2-aminoterephthalic acid； PDA： polydopamine； UHPLC-MS/MS： ultra-high-performance liquid chromatography-tandem mass spectrometry.

### 2.2 生物医药领域

在生物医药分析中，样品前处理会遇到基质干扰大、目标物浓度低、样品体积小等多重挑战^［73，74］^。血浆、尿液等生物样品含有较多的蛋白质和盐分，用传统的固相萃取柱容易堵塞，操作时间长、溶剂用量大，不能满足高效处理的要求^［75，76］^。Fe_3_O_4_/MOF复合材料以分散方式直接与微量生物样品混合，依靠外加磁场快速分离，不需要离心或者过滤^［77，78］^。另外，MOFs的孔道限域效应以及表面功能位点可以对目标药物分子进行选择性富集^［79］^。如图6所示，为了满足生物样品中对某种药物的高选择性捕获要求，Fathi等^［80］^采用逐层自组装法合成了MIL-156@Fe_3_O_4_磁性MOF，然后在其中包覆了共价有机框架（COF），最后在表面包覆了分子印迹聚合物（MIP）层，制备出了MIP@COF@MIL-156@Fe_3_O_4_复合吸附材料。用MSPE法对血浆、尿液样品中孟鲁司特进行选择性富集，用LC-MS/MS进行检测。该策略用MIP层对材料进行特异性识别，适合单一目标物的灵敏检测。当需要同时对血样中的多种抗真菌药物进行萃取时，单一吸附剂的萃取通量会受到限制，Madadkar等^［81］^用碳化NiFeP-MOF@SiO_2_@Fe_3_O_4_多孔纳米花作吸附剂，涡旋辅助萃取后用LC-MS/MS检测，酮康唑、克霉唑、咪康唑的检出限分别为0.3、0.05、0.08 μg/L，相对回收率为92.6%~109.8%。碳化处理产生的多孔结构不但增大了材料的比表面积和传质速率，而且提高了对多种结构相似药物同时捕获的能力，为复杂的生物样品中多残留的分析提供了一种新的方法。生物样品中内源性低丰度磷酸肽的分析也存在着严重的离子抑制问题，使得信号很难被检测出来，对富集材料的亲和力以及排阻能力的要求也更高。本课题组^［23］^在Fe_3_O_4_空心纳米花表面负载UiO-66-NH_2_，并用Zr^4+^修饰后，利用Zr^4+^和磷酸基团之间的亲和力以及多级孔结构，在β-酪蛋白和牛血清白蛋白物质的量之比为1∶1 000的混合体系中仍然可以特异性捕获磷酸肽。用基质辅助激光解吸电离飞行时间质谱（MALDI-TOF MS）进行检测，检出限低至1 fmol，从人血清、唾液中分别检出4条和21条内源性磷酸肽，为低丰度生物标志物的发现提供了一种有效的手段。

**图6:**
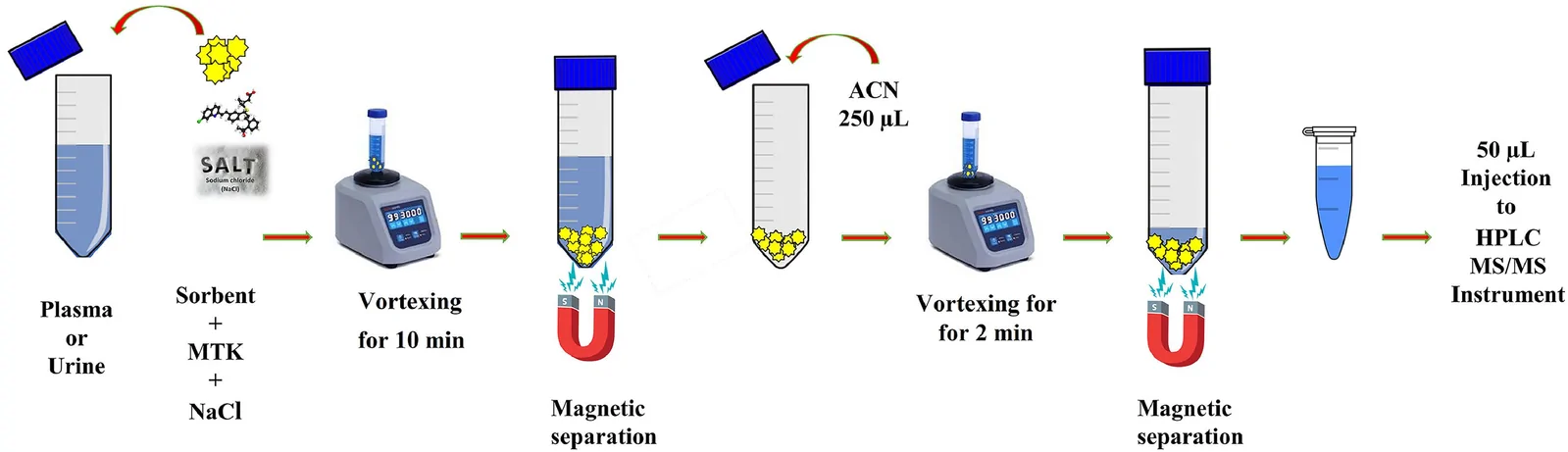
逐层自组装法制备MIP@COF@MIL-156@Fe_3_O_4_复合吸附材料，用于磁分散固相萃取血浆和尿液中孟鲁司特的示意图^［80］^ ACN： acetonitrile； HPLC-MS/MS： high-performance liquid chromatography-tandem mass spectrometry.

### 2.3 环境领域

环境样品中目标污染物浓度低，常常和腐殖酸、悬浮颗粒等共存物结合在一起，单靠过滤或者离心很难有效地分离出来^［79，82］^。Fe_3_O_4_/MOF复合材料的设计可有效解决上述问题，Fe_3_O_4_提供磁响应，使吸附剂可以从浑浊水样中直接回收，避免了传统固相萃取柱堵塞的风险，而MOFs的多孔结构则保证了对各种污染物的高容量捕获^［83，84］^。环境水体中污染物的极性、电荷、分子尺寸等存在较大差别，Fe_3_O_4_/MOF可以通过调节金属节点、有机配体或者添加功能基团等方式改变吸附选择性，不需要更换整个萃取平台^［85，86］^。以磁分离为基础，以MOF结构为可调单元的设计，使得同一种材料可以适应不同的环境基质前处理的要求，也符合绿色分析化学对于溶剂减量、材料重复使用的基本要求。如图7所示，针对六价铬毒性强且共存离子干扰严重的处理需求，Feng等^［87］^以废弃浒苔制备的硫改性KOH活化生物炭（SKBC）为基底，负载Fe_3_O_4_和Cu-MOF，制备出Cu-MOF/Fe_3_O_4_/SKBC复合材料，对Cr（Ⅵ）的最大吸附容量达227.95 mg/g。该材料中的Fe^2+^、Cu^2+^可以把Cr（Ⅵ）还原成低毒的Cr（Ⅲ），吸附和还原同时进行，Fe_3_O_4_还具有快速磁分离的能力，20 s就能完成固液分离，经过5次循环之后，去除率仍然可以达到90%以上。在实现高效去除的基础上，进一步降低检出限和样品用量成为环境分析的目标。Uzcan和Soylak^［88］^以Fe_3_O_4_@MIL‑53（Fe）@Ti_3_AlC_2_为吸附剂，pH 5.0条件下涡旋吸附30 s，磁铁快速分离后加入0.5 mL 3 mol/L HNO_3_涡旋洗脱2 min，再次磁分离后取洗脱液直接进样，采用高分辨连续光源火焰原子吸收光谱测定Cd（Ⅱ）含量。该方法不需要离心，检出限为3.6 ng/mL，富集倍数为40倍，吸附剂可以重复使用6次。相比于MSPE，磁分散固相微萃取（MD-μ-SPE）将样品体积降低到20 mL以下，洗脱体积减少到0.5 mL左右，大大提高了富集倍数和检测灵敏度。为了提高传质效率、萃取速度，Ozalp等^［89］^用水热法和溶胶-凝胶法制备了MOF-74（Co）@Fe_3_O_4_纳米复合材料，用微电机驱动的MD-μ-SPE技术对富集后的水样和食品中Pb（Ⅱ）、Cu（Ⅱ）进行分离和富集。该策略用H_2_O_2_作为燃料，在材料表面催化分解产生氧气气泡来驱动微电机运动，主动提高吸附剂和目标离子的接触效率，克服了传统被动扩散的传质限制。优化条件下Pb（Ⅱ）、Cu（Ⅱ）的检出限分别为1.90 μg/L、0.27 μg/L，富集因子分别为77、79，在高浓度干扰离子存在时仍能获得95%以上的回收率，可以用于实际水样中Pb（Ⅱ）、Cu（Ⅱ）的检测。

**图7:**
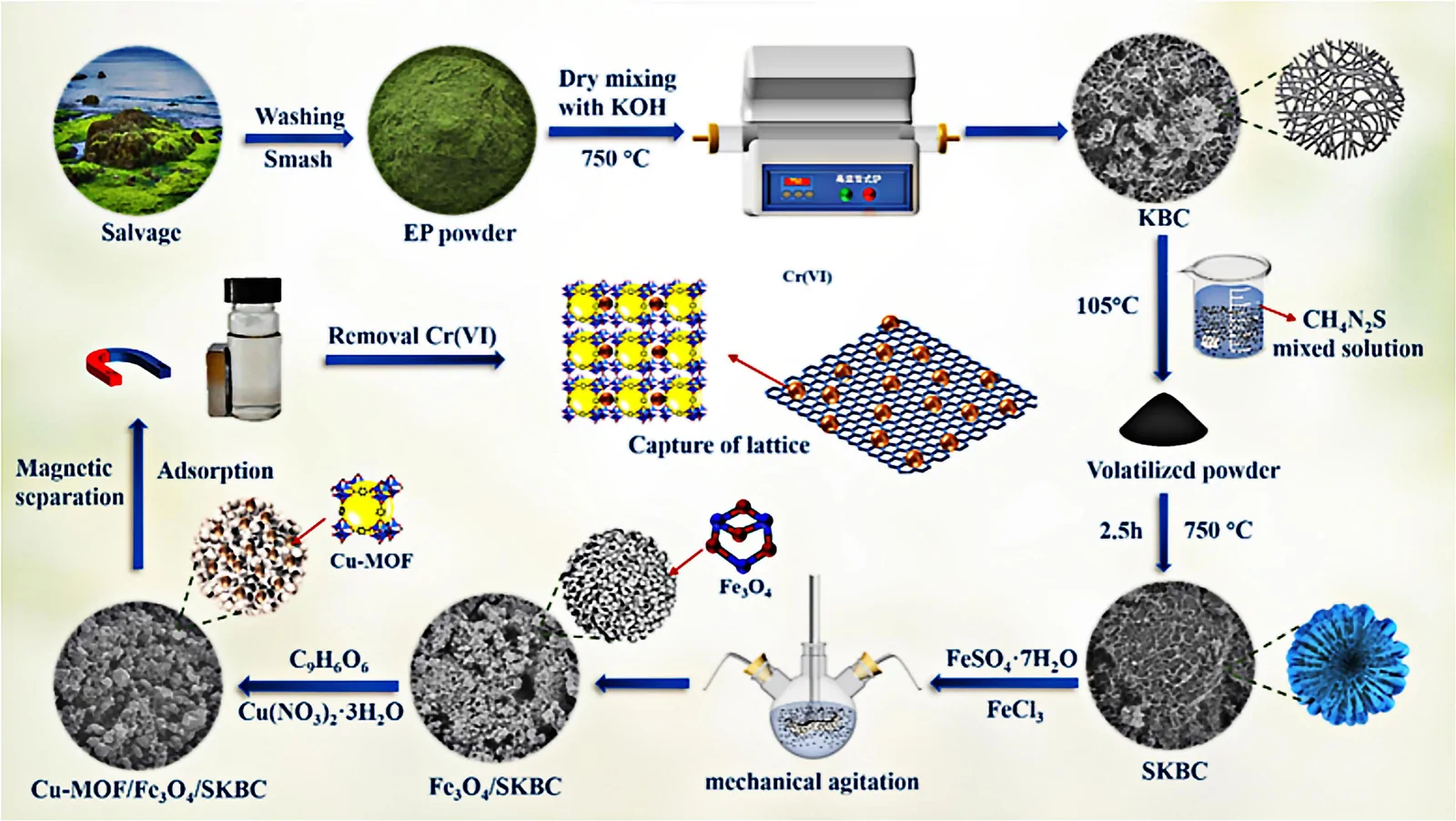
Cu-MOF/Fe_3_O_4_/SKBC复合材料对Cr（Ⅵ）的吸附-还原协同去除及磁性固相萃取示意图^［87］^ EP： *Enteromorpha* prolifera； KBC： KOH-activated *Enteromorpha* biochar； SKBC： sulfur-modified KOH-activated *Enteromorpha* biochar； Cu-MOF： copper-based metal-organic framework.

### 2.4 其他领域

Fe_3_O_4_/MOF复合材料在化妆品安全检测、法医毒理学等领域也有着应用前景。化妆品基质含有油脂、表面活性剂、色素和防腐剂等成分，传统的前处理方法步骤烦琐且有机溶剂消耗大^［90］^。Li等^［91］^制备了类石墨氮化碳（g-C_3_N_4_）功能化的磁性MOF复合材料（Fe_3_O_4_/g-C_3_N_4_/MIL-101），用于化妆品中5种糖皮质激素的MSPE富集，g-C_3_N_4_的加入使MIL-101具有更好的水稳定性以及选择性。结合UPLC-MS/MS检测，检出限为2 ng/L，回收率为77.3%~113%，吸附剂可重复使用9次。Jalilian等^［92］^ 将磁铁矿与多壁碳纳米管复合成磁性多壁碳纳米管（MMWCNTs），再与MIL-101（Cr）复合，制得MMWCNTs@MIL-101（Cr）。多壁碳纳米管的加入不但增大了比表面积，而且有效抑制了Fe_3_O_4_纳米颗粒的团聚。而MIL-101（Cr）本身在水相中稳定性较好，三者协同作用使吸附剂在萃取对羟基苯甲酸酯、邻苯二甲酸酯时具有较快的动力学（13.5 min）和较高的富集倍数（最高可达70.6）。用响应面法优化了萃取条件，检出限为0.03～0.15 μg/L，用于膏霜类化妆品和水样的分析时，加标回收率为74%～97%。该工作将磁性碳纳米管/MOF复合物用于对羟基苯甲酸酯和邻苯二甲酸酯两类化合物的同步萃取，为复杂基质中多残留同时检测提供了一种可能的方法。Zac等^［93］^采用磁性固相萃取比较了Fe_3_O_4_@MIL-101（Cr）、Fe_3_O_4_@MIL-101（Cr）@NH_2_和Fe_3_O_4_@MIL-101（Cr）@SO_3_H 3种吸附剂对9种对羟基苯甲酸酯的萃取性能，结果表明氨基修饰的Fe_3_O_4_@MIL-101（Cr）@NH_2_萃取效率最高。该材料主要通过氨基与对羟基苯甲酸酯酚羟基之间的氢键作用以及MOF骨架的*π-π*堆积作用协同富集目标物。整个萃取仅需6 min，结合LC-MS/MS检测，检出限低至0.063~0.622 ng/mL。将该方法应用于辣椒粉、面部爽肤水实际样品中，各检测出8种、4种对羟基苯甲酸酯，加标回收率为72.9%～108%、85.1%～104%；相比于文献中报道的其他MOF基吸附剂如HKUST-1、MIL-53（Al）泡沫等，该研究所得的材料在萃取时间及检出限上均有优势。法医毒理学中生物样品基质复杂、目标毒物浓度低，对样品前处理方法的灵敏度和选择性要求很高^［94，95］^。Zhang等^［96］^以氨基硅烷为连接臂，将Fe_3_O_4_纳米粒子锚定在钴基腺嘌呤MOF（bio-MOF-1）表面，制备了磁性生物MOF复合材料（Fe_3_O_4_-NH_2_/bio-MOF-1），用于尿样和废水中6种苯二氮䓬类药物的MDSPE富集，结合LC-MS进行检测。该材料依靠静电吸引、*π-π*堆积和氢键三者共同作用，在最佳情况下检出限为0.71~2.49 ng/L，加标回收率为80.2%~94.5%，重复使用10次后性能没有明显下降。在此基础上，Abad等^［97］^又提出了MOF-on-MOF异质结构来提高吸附量和萃取效率。通过溶胶-凝胶策略合成Fe_3_O_4_@SiO_2_@Co-MOF@Fe-MOF磁性复合材料，用于人尿中地西泮和劳拉西泮的MD-μ-SPE，结合高效液相色谱-紫外检测法（HPLC-UV）检测。分级孔结构以及更多的暴露活性位点使两种药物的检出限分别为0.09 ng/mL和0.06 ng/mL，富集因子分别为654和719，加标回收率为88.2%~97.5%。Zhang等^［98］^制备了羧基功能化的磁性锆基MOF（Fe_3_O_4_@SiO_2_@UiO-67-COOH），利用UiO-67的孔径筛分效应在萃取抗精神病药物的同时直接排除血清中98.9%~99.8%的蛋白质，省去了烦琐的蛋白沉淀步骤。将该材料和96孔自动提取仪联合使用，9 min内就可以完成96个血清样本的自动化磁固相萃取，结合UPLC-MS/MS检测，7种药物及其代谢物的检出限为0.06~0.90 ng/mL，加标回收率为95.7%~112.3%，重复使用3次后回收率仍大于84.5%。

## 3 结论与展望

Fe_3_O_4_/MOF复合材料兼具高效富集和快速磁分离能力，已在多类复杂样品的前处理中得到应用。与传统吸附材料相比，该材料在富集效率、操作便捷性及与高灵敏检测技术的适配性方面具有优势，已用于食品、生物、环境、化妆品及法医毒理学样品的前处理。目前报道的制备方法仍各有局限：一锅法容易造成MOF负载不均、形貌不可控，原位生长法与逐层自组装法反应条件苛刻、操作复杂；后合成修饰法虽然可以引入功能基团，但是多步反应会降低材料的稳定性。复合材料普遍存在着循环使用次数少、在复杂基质中长期性能验证不足等状况。针对上述问题，未来研究可从制备过程、材料功能和分析模式3个方面推进。首先，可采用微流控技术进行连续制备工艺的研究。在受限空间中控制成核和生长过程，可以获得粒径分布窄、MOF壳层厚度均匀的复合材料，提高材料制备的批次稳定性。其次，可构建具有自清洁功能的智能型Fe_3_O_4_/MOF吸附剂，通过引入光响应、pH响应或者热响应的智能涂层，吸附完成后通过外界刺激触发表面分子的构象变化或者脱落，从而减少洗脱步骤和溶剂消耗。此外，可通过设计介孔-大孔分级孔结构并结合抗体、适配体等亲和配体进行定向修饰，拓展其在低丰度大分子生物标志物富集中的应用。最后，推进前处理过程与检测仪器的在线联用，是提升方法自动化水平并促进实际应用的重要方向。

## References

[1] Rezaei H，Matin A A，Soylak M . Talanta，2026，304： 129546 10.1016/j.talanta.2026.129546 41707524

[2] Zhou H，Yuan Y，Liu Z，et al . TrAC-Trends Anal Chem，2025，191： 118355

[3] Ali A，Qin T，Zhang W，et al . Anal Chim Acta，2025，1355： 343934 10.1016/j.aca.2025.343934 40274337

[4] Song X，Li F，Yan T，et al . Process Saf Environ Prot，2022，165： 610

[5] Wang X，Cui J，Sun L，et al . Food Anal Methods，2024，17（4）： 534

[6] Yao T，Song J，Zhou C，et al . Sep Purif Technol，2024，341： 126979

[7] Mu M，Zhu S，Gao Y，et al . Talanta，2023，260： 124540 10.1016/j.talanta.2023.124540 37116361

[8] Mohiuddin I，Bhogal S，Gupta K . Talanta，2026，296： 128519 10.1016/j.talanta.2025.128519 40582105

[9] Chen R，Qiao X，Liu F . Anal Chim Acta，2022，1201： 339632 10.1016/j.aca.2022.339632 35300789

[10] Huang Y，Ma C，Huang X . J Chromatogr A，2025，1742： 465653 10.1016/j.chroma.2025.465653 39765204

[11] Liu X，Du Y，Mourdikoudis S，et al . Adv Opt Mater，2023，11（18）： 2202859

[12] Manouchehri M，Seidi S，Abdullah F O . Talanta，2021，229： 122273 10.1016/j.talanta.2021.122273 33838775

[13] Kang Y，Shi S，Sun H，et al . J Agric Food Chem，2022，70（51）： 16050 10.1021/acs.jafc.2c07132 36533981

[14] Li Z，Yang F，Yin Y . Adv Funct Mater，2019，30（2）： 1903467

[15] Karvelas E G，Lampropoulos N K，Benos L T，et al . Comput Methods Programs Biomed，2021，198： 105778 10.1016/j.cmpb.2020.105778 33039920

[16] Liu S，Yu B，Wang S，et al . Adv Colloid Interface Sci，2020，281： 102165 10.1016/j.cis.2020.102165 32361408

[17] Rudravarapu K，Yarramuthi V，Munagapati V S，et al . Inorg Chem Commun，2025，182： 115588

[18] Li W，Xu P，Wang Z，et al . Mater Chem Front，2023，7（21）： 5140

[19] Schneider C，Bodesheim D，Keupp J，et al . Nat Commun，2019，10（1）： 4921 10.1038/s41467-019-12876-1 PMC6820732 31664026

[20] Yang J，Zhou H，Hao Y，et al . J Chromatogr A，2025，1747： 465814 10.1016/j.chroma.2025.465814 40014963

[21] Tian X，Li Y，Ren M，et al . J Hazard Mater，2026，501： 140733 10.1016/j.jhazmat.2025.140733 41371141

[22] Abednatanzi S，Gohari Derakhshandeh P，Depauw H，et al . Chem Soc Rev，2019，48（9）： 2535 10.1039/c8cs00337h 30989162

[23] Zhang N，Qin M，Zhu J，et al . Chin Chem Lett，2025，36（4）： 110177

[24] Lou X，Hao Y，Zhou H，et al . J Chromatogr A，2025，1742： 465656 10.1016/j.chroma.2025.465656 39778283

[25] Zhang S，Ding J，Tian D，et al . J Solid State Chem，2024，336： 124769

[26] Vállez-Gomis V，Grau J，Benedé J L，et al . TrAC-Trends Anal Chem，2024，171： 117486

[27] Maya F，Palomino Cabello C，Frizzarin R M，et al . TrAC-Trends Anal Chem，2017，90： 142

[28] Qin M，Li D，Zhou M，et al . J Hazard Mater，2025，495： 138952 10.1016/j.jhazmat.2025.138952 40554336

[29] Li D，Qin M，Lou X，et al . J Chromatogr A，2024，1737： 465463 10.1016/j.chroma.2024.465463 39490196

[30] Chaouiki A，Chafiq M，Salghi R，et al . Prog Mater Sci，2026，158： 101638

[31] Li S，Ma J，Cheng J，et al . TrAC-Trends Anal Chem，2023，168： 117296

[32] Zhang S，Su W，Peng C，et al . Mater Sci Eng B，2026，324： 118974

[33] Zhang S，Ding J，Tian D，et al . J Mol Struct，2024，1300： 137313

[34] Qin M，Li D，Zhu J，et al . J Chromatogr A，2024，1726： 464961 10.1016/j.chroma.2024.464961 38723491

[35] Li L，Wang H，Fang J . ACS Appl Mater Interfaces，2024，16（32）： 42623 10.1021/acsami.4c07120 39090771

[36] Wen H，Xu W，Lin S，et al . Carbon，2024，230： 119613

[37] Mourdikoudis S，Dutta S，Kamal S，et al . Adv Mater，2025，37（52）： 2415399 10.1002/adma.202415399 PMC12747499 40255059

[38] Wang Q，Chen L，Cui X，et al . Talanta，2023，256： 124268 10.1016/j.talanta.2023.124268 36657241

[39] Huang G，Pan Z，Wang Q，et al . Anal Chim Acta，2026，1405： 345469 10.1016/j.aca.2026.345469 42009420

[40] Soylak M，Uzcan F，Goktas O，et al . Food Chem，2023，429： 136855 10.1016/j.foodchem.2023.136855 37478612

[41] Ahmadi Y，Ramazani A，Mousavi S M . J Organomet Chem，2025，1038： 123753

[42] Ma Y，Xu G，Wei F，et al . ACS Appl Mater Interfaces，2018，10（24）： 20801 10.1021/acsami.8b05643 29856924

[43] Wang Z，Han L，Xia Q，et al . ACS Appl Nano Mater，2025，8（37）： 18098

[44] Chong X，Bao W，Wang Y，et al . J Solid State Chem，2025，348： 125390

[45] Abdtawfeeq T H，Farhan Z A，Al-Majdi K，et al . J Inorg Organomet Polym Mater，2022，33（2）： 472

[46] Kong M，Liu X，Jia Z，et al . J Colloid Interface Sci，2021，604： 39 10.1016/j.jcis.2021.07.003 34261018

[47] Liang X，Wu Y，Chen G，et al . J Phys： Mater，2026，9（1）： 012004

[48] Xiang X，Mao X，Ding X，et al . J Hazard Mater，2024，473： 134588 10.1016/j.jhazmat.2024.134588 38797072

[49] Feng S，Zhang A，Wu F，et al . Anal Chim Acta，2022，1206： 339772 10.1016/j.aca.2022.339772 35473877

[50] Li B，Liao X，Chen Y . J Hazard Mater，2025，496： 139373 10.1016/j.jhazmat.2025.139373 40753804

[51] Mosupi K，Masukume M，Weng G，et al . Coord Chem Rev，2025，529： 216467

[52] Miyah Y，El Messaoudi N，Benjelloun M，et al . Mater Today Sustainability，2024，28： 100985

[53] Haghighat H . Microchem J，2022，181： 107770

[54] Li J-Y，Zhang S，Gao W，et al . ACS Sustainable Chem Eng，2020，8（44）： 16422

[55] Yazdiamiri F，Alinezhad H，Kiani A，et al . Sci Rep，2025，15（1）： 37267 10.1038/s41598-025-21356-0 PMC12552603 41136739

[56] Zhu K，Xu M，Yang Q，et al . Nanoscale，2026，18（8）： 4215 10.1039/d5nr04596g 41610440

[57] Yadav S，Dixit R，Sharma S，et al . Mater Adv，2021，2（7）： 2153

[58] He Q，Bai J，Wang H，et al . Small，2023，19（50）： 2303884 10.1002/smll.202303884 37625077

[59] Lu Y，Zhou H，Yang H，et al . J Mater Chem A，2024，12（11）： 6243

[60] Gao Y，Zhu S，Mu M，et al . Chem Eng J，2023，475： 146459

[61] Karaket R，Detsri E，Khattiya A，et al . J Chromatogr A，2024，1717： 464668 10.1016/j.chroma.2024.464668 38278132

[62] Huang J，Li J，Lu C，et al . J Environ Health Sci Eng，2025，23（2）： 34 10.1007/s40201-025-00956-y PMC12515188 41084722

[63] Datta D，Biswas B，Lodh A，et al . Talanta，2025，293： 128094 10.1016/j.talanta.2025.128094 40239584

[64] Mohan B，Virender V，Raghav N，et al . Microchem J，2026，221： 117043

[65] Duo H，Yue J，Zhang X，et al . Microchem J，2024，196： 109557

[66] Li X，Du L，Si T，et al . TrAC-Trends Anal Chem，2026，198： 118744

[67] Xu C-Y，Cui Y-Y，Yang C-X . TrAC-Trends Anal Chem，2025，185： 118171

[68] Wang Q，Gao T，Hao L，et al . TrAC-Trends Anal Chem，2020，132： 116048

[69] Feng J，Dong Y，Li H，et al . J Hazard Mater，2025，495： 139040 10.1016/j.jhazmat.2025.139040 40570626

[70] Lv L，Liu H，Wang L，et al . Food Chem，2025，486： 144604 10.1016/j.foodchem.2025.144604 40345031

[71] Zhang S，Li Y，Wang Y，et al . J Water Process Eng，2025，74： 107738

[72] Jiang X，Cheng J，Du Q，et al . Food Control，2023，154： 110035

[73] Cui Y，Ding L，Ding J . TrAC-Trends Anal Chem，2022，147： 116514

[74] Wang M，Cheng Y，Zhang M，et al . J Pharm Anal，2026： 101543 10.1016/j.jpha.2025.101543 PMC13499175 42633210

[75] Nikkhoo E，Dinari M，Lützenkirchen J，et al . Adv Colloid Interface Sci，2026，354： 103898 10.1016/j.cis.2026.103898 41967289

[76] Chen R，Bao Y，Huang R，et al . Biomaterials，2026，333： 124201 10.1016/j.biomaterials.2026.124201 41966551

[77] Aguirre M Á，Canals A . TrAC-Trends Anal Chem，2022，146： 116500

[78] Castañeda F N，Prince D L，Peirano S R，et al . TrAC-Trends Anal Chem，2024，180： 117924

[79] Vega-Morales T，Sosa-Ferrera Z，Santana-Rodríguez J J，et al . TrAC-Trends Anal Chem，2025，183： 118116

[80] Fathi A A，Afshar Mogaddam M R，Sorouraddin S M，et al . J Chromatogr A，2024，1738： 465478 10.1016/j.chroma.2024.465478 39509854

[81] Madadkar N，Seidi S，Abdullah F O，et al . Talanta，2026，301： 129333 10.1016/j.talanta.2025.129333 41485405

[82] Gao Y，Liu G，Gao M，et al . Crit Rev Anal Chem，2019，50（5）： 472 10.1080/10408347.2019.1653166 31423806

[83] Mbuyazi T B，Ajibade P A . Discover Nano，2024，19（1）： 158 10.1186/s11671-024-04102-9 PMC11438764 39342049

[84] Grbic J，Nguyen B，Guo E，et al . Environ Sci Technol Lett，2019，6（2）： 68

[85] González N，Aguinaga Martínez M V，Domini C E，et al . Trends Environ Anal Chem，2023，37： e00197

[86] Gamal M，Imam M S，Albugami A S，et al . Trends Environ Anal Chem，2024，43： e00236

[87] Feng S，Lu Y，Yang L，et al . Sep Purif Technol，2026，389： 136834

[88] Uzcan F，Soylak M . J Food Compos Anal，2024，135： 106636

[89] Ozalp O，Soylak M . J Ind Eng Chem，2026，157： 796

[90] Salamat Q，Özen M B，Soylak M . Microchem J，2025，208： 112286

[91] Li Y，Chen X，Xia L，et al . J Chromatogr A，2019，1606： 460382 10.1016/j.chroma.2019.460382 31353072

[92] Jalilian N，Ebrahimzadeh H，Asgharinezhad A A . J Chromatogr A，2019，1608： 460426 10.1016/j.chroma.2019.460426 31416622

[93] Zac R A T，Jung W-T，Kuo Y-J，et al . J Chin Chem Soc，2024，71： 763

[94] El-Deen A K，Hussain C M . Trends Environ Anal Chem，2023，39： e00209

[95] Leszczyńska D，Hallmann A，Treder N，et al . Talanta，2024，270： 125613 10.1016/j.talanta.2023.125613 38159351

[96] Zhang S，Yao W，Fu D，et al . J Sep Sci，2018，41（8）： 1864 10.1002/jssc.201701226 29356344

[97] Abad M O K，Masrournia M，Javid A . Microchem J，2024，197： 109797

[98] Zhang Z，Han W，Qing J，et al . J Hazard Mater，2024，465： 133189 10.1016/j.jhazmat.2023.133189 38071772

